# Further Evidence That OPG rs2073618 Is Associated With Increased Risk of Musculoskeletal Symptoms in Patients Receiving Aromatase Inhibitors for Early Breast Cancer

**DOI:** 10.3389/fgene.2021.662734

**Published:** 2021-06-15

**Authors:** Daniel L. Hertz, Karen Lisa Smith, Yuhua Zong, Christina L. Gersch, Andrea M. Pesch, Jennifer Lehman, Amanda L. Blackford, N. Lynn Henry, Kelley M. Kidwell, James M. Rae, Vered Stearns

**Affiliations:** ^1^Department of Clinical Pharmacy, University of Michigan College of Pharmacy, Ann Arbor, MI, United States; ^2^Sidney Kimmel Comprehensive Cancer Center, Johns Hopkins School of Medicine, Baltimore, MD, United States; ^3^Department of Biostatistics, University of Michigan School of Public Health, Ann Arbor, MI, United States; ^4^Department of Internal Medicine, Division of Hematology/Oncology, University of Michigan Medical School, Ann Arbor, MI, United States

**Keywords:** Pharmacogenetics, aromatase inhibitor, Musculoskeletal adverse events, *OPG*, *TCL1A*, breast cancer

## Abstract

**Background:**

Aromatase inhibitors (AI) reduce recurrence and death in patients with early-stage hormone receptor-positive (HR +) breast cancer. Treatment-related toxicities, including AI-induced musculoskeletal symptoms (AIMSS), are common and may lead to early AI discontinuation. The objective of this study was to replicate previously reported associations for candidate germline genetic polymorphisms with AIMSS.

**Methods:**

Women with stage 0-III HR + breast cancer initiating adjuvant AI were enrolled in a prospective clinic-based observational cohort. AIMSS were assessed by patient-reported outcomes (PRO) including the PROMIS pain interference and physical function measures plus the FACT-ES joint pain question at baseline and after 3 and 6 months. For the primary analysis, AIMSS were defined as ≥ 4-point increase in the pain interference T-score from baseline. Secondary AIMSS endpoints were defined as ≥ 4-point decrease in the physical function T-score from baseline and as ≥ 1-point increase on the FACT-ES joint pain question from baseline. The primary hypothesis was that *TCL1A* rs11849538 would be associated with AIMSS. Twelve other germline variants in *CYP19A1*, *VDR*, *PIRC66*, *OPG*, *ESR1*, *CYP27B1*, *CYP17A1*, and *RANKL* were also analyzed assuming a dominant genetic effect and prespecified direction of effect on AIMSS using univariate logistic regression with an unadjusted α = 0.05. Significant univariate associations in the expected direction were adjusted for age, race, body mass index (BMI), prior taxane, and the type of AI using multivariable logistic regression.

**Results:**

A total of 143 participants with PRO and genetic data were included in this analysis, most of whom were treated with anastrozole (78%) or letrozole (20%). On primary analysis, participants carrying *TCL1A* rs11849538 were not more likely to develop AIMSS (odds ratio = 1.29, 95% confidence interval: 0.55–3.07, *p* = 0.56). In the statistically uncorrected secondary analysis, *OPG* rs2073618 was associated with AIMSS defined by worsening on the FACT-ES joint pain question (OR = 3.33, *p* = 0.004), and this association maintained significance after covariate adjustment (OR = 3.98, *p* = 0.003).

**Conclusion:**

Carriers of *OPG* rs2073618 may be at increased risk of AIMSS. If confirmed in other cohorts, *OPG* genotyping can be used to identify individuals with HR + early breast cancer in whom alternate endocrine therapy or interventions to enhance symptom detection and implement strategies to reduce musculoskeletal symptoms may be needed.

## Introduction

Adjuvant endocrine therapy for 5–10 years reduces recurrence and death after early-stage hormone receptor positive (HR +) breast cancer. Based on multiple trials demonstrating improved breast cancer outcomes when compared to treatment with adjuvant tamoxifen, a third-generation aromatase inhibitor (AI) (anastrozole, letrozole, or exemestane) is generally the preferred adjuvant endocrine therapy for postmenopausal women with early stage HR + breast cancer (Francis et al., 2018). Additionally, the use of an adjuvant AI in conjunction with ovarian suppression or ablation in high-risk premenopausal women with HR + breast cancer is associated with improved breast cancer outcomes (Hadji et al., 2013). The AI works by inhibiting the aromatase (CYP19A1) enzyme that is responsible for converting androgens to estrogens. This depletes the body of estrogens and starves the tumor of the estrogenic growth factor causing cellular replication.

Despite the established benefits of adjuvant endocrine therapy, up to 50% of patients are non-adherent or discontinue their treatment early, with some studies demonstrating higher rates of early discontinuation with AI therapy compared to tamoxifen (Partridge et al., 2003, 2008; Crew et al., 2007; Hershman et al., 2010, 2011, 2016, 2020; Henry et al., 2012, 2017; Murphy et al., 2012; Kemp et al., 2014; Kadakia et al., 2016). Risks of breast cancer recurrence and death are higher among individuals who are non-adherent to or who discontinue adjuvant endocrine therapy early (Kuba et al., 2016). Determinants of AI non-adherence and early discontinuation are multifactorial and include both baseline and treatment-emergent symptoms, especially AI-induced musculoskeletal symptoms (AIMSS) (Partridge et al., 2003, 2008; Felson and Cummings, 2005; Henry et al., 2012; Murphy et al., 2012; Chim et al., 2013; Bender et al., 2014; Kemp et al., 2014; Kidwell et al., 2014; Hershman et al., 2016; Neugut et al., 2016; Nabieva et al., 2018a,b; Wagner et al., 2018; Wulaningsih et al., 2018; Shinn et al., 2019; Wheeler et al., 2019).

Up to 50% of patients with breast cancer treated with adjuvant AI therapy experience AIMSS, a syndrome characterized by symptoms including joint pain and stiffness, myalgias, carpal tunnel syndrome, tenosynovitis, and/or reduced grip strength that is thought to be attributable to estrogen deprivation (Baum et al., 2003; Coates et al., 2007; Mao et al., 2009; Hershman et al., 2011; Hadji et al., 2014; Moscetti et al., 2015; Beckwée et al., 2017; Gupta et al., 2020). Among patients who discontinue adjuvant AI therapy due to symptoms or side effects, AIMSS are a leading reason for discontinuation (Felson and Cummings, 2005; Partridge et al., 2008; Murphy et al., 2012; Olufade et al., 2015). Musculoskeletal pain is associated with lower health-related quality of life in breast cancer patients receiving adjuvant AI therapy (Sitlinger et al., 2019), and endocrine therapy side effects, including pain, are associated with limitations of physical function (Sestak et al., 2008). Evidence-based interventions to manage AIMSS include strategies such as exercise, yoga, duloxetine, and acupuncture (Moscetti et al., 2015). Clinicians may also consider transitioning patients with AIMSS from one AI to another or switching to tamoxifen (Moscetti et al., 2015).

To date, accurate clinical predictors of AIMSS have not been identified. While some studies have demonstrated associations between breast cancer stage, body mass index (BMI), prior chemotherapy (especially taxanes), prior tamoxifen, and time since last menstrual period with AIMSS in postmenopausal women receiving AI therapy for early-stage HR + breast cancer, these associations have not been consistent across studies (Baum et al., 2003; Hershman et al., 2011; Hertz et al., 2017; Gupta et al., 2020). By identifying individuals at highest risk for AIMSS for enhanced symptom monitoring and management, an accurate pretreatment predictor of AIMSS has the potential not only to reduce pain and improve quality of life but also to improve endocrine therapy adherence and reduce early endocrine therapy discontinuation, thereby improving breast cancer outcomes.

Several studies have investigated potential physiological biomarkers for AIMSS including germline genetics [reviewed by Hertz et al. (2017)]. Many studies have used candidate-gene or candidate-single nucleotide polymorphism (SNP) approaches to investigate whether inherited germline variants affect AIMSS risk (Ingle et al., 2010; Park et al., 2011; Garcia-Giralt et al., 2013; Henry et al., 2013; Wang et al., 2013, 2015; Fontein et al., 2014; Leyland-Jones et al., 2015; Lintermans et al., 2016; Dempsey et al., 2018). These studies have reported several discovery-phase associations including inherited polymorphism in the genes that encode for the AI drug target CYP19A1 (Garcia-Giralt et al., 2013; Henry et al., 2013; Fontein et al., 2014; Leyland-Jones et al., 2015), proteins responsible for bone resorption *OPG*/*RANKL* (Wang et al., 2013; Lintermans et al., 2016; Dempsey et al., 2018), and the estrogen receptor *ESR1* (Ingle et al., 2010; Park et al., 2011). In addition, a hypothesis-agnostic genome-wide association study of the MA.27 clinical trial comparing anastrozole and exemestane reported that patients who carried rs11849538 near *TCL1A* had increased AIMSS risk (Liu et al., 2012). Although this association has not been successfully replicated, the well-done discovery and mechanistic support (Snyder et al., 2009) justifies further attempts to replicate the putative association in independent cohorts of AI-treated patients.

In order to pursue clinical translation of any of these potential genetic predictors of AIMSS, additional replication and retrospective validation of their association with AIMSS in independent patient cohorts is necessary. The primary objective of this study was to replicate the association of *TCL1A* rs11849538 with patient-reported AIMSS during the first 6 months of AI therapy in a prospectively accrued cohort of patients with early-stage HR + breast cancer initiating adjuvant AI therapy. The secondary objective was to utilize this cohort to attempt replication for other candidate SNPs that have been previously reported to be associated with AIMSS.

## Materials and Methods

### Patients and AIMSS Data

This was a pharmacogenetic analysis of women with stage 0-III HR + breast cancer initiating adjuvant endocrine therapy who enrolled in a prospective observational clinic-based cohort at the Sidney Kimmel Comprehensive Cancer Center at Johns Hopkins from March 2012 through December 2016 (ClinicalTrials.gov Identifier: NCT01937052). Selection of the endocrine therapy regimen (tamoxifen versus AI with or without ovarian suppression) was determined by the treating clinician and recorded in the study database. Participants completed patient-reported outcome (PRO) measures assessing a range of symptom domains online using the PatientViewpoint interface at baseline and at 3, 6, 12, 24, 36, 48, and 60 months (Garcia et al., 2007; Snyder et al., 2013; Wu et al., 2016). This study was approved by the Johns Hopkins IRB, and all participants completed written informed consent prior to enrollment.

Participants included in this secondary analysis received a third-generation AI (anastrozole, letrozole, or exemestane) and completed the PRO measures at baseline and at 3 and/or 6 months. PRO measures related to AIMSS included the PROMIS pain interference and physical function questionnaires (Fallowfield et al., 1999; Jensen et al., 2017a) and a single item on the FACT-ES questionnaire addressing joint pain. The FACT-ES includes 19 items addressing a range of endocrine symptoms, including joint pain, to which respondents report symptom severity on a 5-point scale ranging from 0 (“not at all”) to 4 (“very much”) (Schalet et al., 2016). PROMIS measures are scored using a T-score metric with 50 representing the mean score in the United States population. Higher scores on PROMIS measures indicate more of the outcome measured (i.e., a higher pain interference T-score indicates more pain interference, and a lower physical function T-score indicates worse physical function). In patients with early-stage cancer, the minimal important difference (MID) on PROMIS measures, determined using a distribution-based method, is three to five points (Fallowfield et al., 1999; Rohlfs and Weir, 2008; Yost et al., 2011; Teresi et al., 2016; Jensen et al., 2017b). For this analysis, we selected the midpoint of the range reported for the MID for the PROMIS measures in patients with early-stage cancer, four points.

Our primary AIMSS endpoint was an increase of four or more points on the PROMIS pain interference T-score from baseline to 3 or 6 months. A secondary AIMSS endpoint was a decrease of four or more points on the PROMIS physical function T-score from baseline to 3 or 6 months. The other secondary AIMSS endpoint was an increase of one or more point on the FACT-ES question, “I have pain in my joints” from baseline to 3 or 6 months (Schalet et al., 2016). For patients who received multiple AIs due to switching treatment, only the data from their first AI treatment were included in the analysis.

### Genotyping

A whole blood or a saliva sample was collected at baseline and stored at −80°C for germline DNA isolation. Germline DNA was isolated from whole blood using DNeasy Blood and Tissue Kits (Qiagen, Valencia, CA, United States) following the manufacturer’s instructions. Germline DNA was isolated from saliva samples using prepIT-L2P (DNA Genotek, ON, Canada) following the manufacturer’s instructions. Thirteen candidate gene variants of interest with a prespecified direction of effect on AIMSS were selected. The *a priori* determined primary analysis was an attempted replication of the increased AIMSS risk for carriers of *TCL1A* rs11849538. The other 12 SNPs and their predetermined direction of effect were tested in a secondary, statistically uncorrected analysis. Genotyping was performed using TaqMan^TM^ Allelic Discrimination assays according to the manufacturer’s instructions (Applied Biosystems, Foster City, CA, United States). The SNP assay IDs used in this study were the following: rs10046 (C___8234731_30), rs11568820 (C___2880808_10), rs11849538 (C___1927667_30), rs16964189 (C__34453639_10), rs2073618 (C___1971047_40), rs2234693 (C___3163590_10), rs4646536 (C__25623453_10), rs6163 (C__12119916_1_), rs7176005 (C_189237142_10), rs7984870 (C__29811035_20), rs9322336 (C__29568677_10), rs9340799 (C___3163591_10), and rs934635 (C___8794643_10). PCRs were carried out using 10 ng of DNA with Genotyping Master Mix (Applied Biosystems) in a CFX96 real-time PCR detection system (Bio-Rad, Madison, WI, United States) for 35 cycles. Genotype quality assurance was assessed by random selection of 10% of DNA samples for re-genotyping, and the results were 100% concordant.

### Statistical Analysis

The univariate association for each genetic predictor assuming a dominant genetic effect with AIMSS risk defined by the three previously described endpoints was tested using logistic regression. Associations were only considered replicated if the direction of effect was consistent with our prespecified expected direction, based on previously reported associations. The primary hypothesis was that patients carrying *TCL1A* rs11849538 would have a higher risk of AIMSS, as defined by the primary endpoint of an increase of four or more points on the PROMIS pain interference measure from baseline to 3 or 6 months. The secondary analyses examined the association of the other 12 candidate variants with AIMSS defined by the pain interference measure and the association of all 13 candidate variants with AIMSS defined by either of the other two AIMSS definitions, for a total of 1 primary analysis and 38 statistically uncorrected secondary analyses. All associations were tested using an α = 0.05, as appropriate for a single primary analysis and exploratory uncorrected secondary analyses. Any significant univariate associations were then corrected for relevant covariates: age in years (as a continuous variable), self-reported race (White versus other), baseline BMI (as a continuous variable), prior taxane chemotherapy (versus non-taxane chemotherapy or no chemotherapy), and aromatase inhibitor (anastrozole versus other due to low frequency of exemestane). In addition, any significant univariate associations assuming a dominant genetic model were then tested assuming recessive and additive genetic effects to identify the genetic model that best explained the association. Statistical analyses were conducted using R version 3.6.3.

## Results

### Patients, AIMSS, and Genetics

**FIGURE 1:**
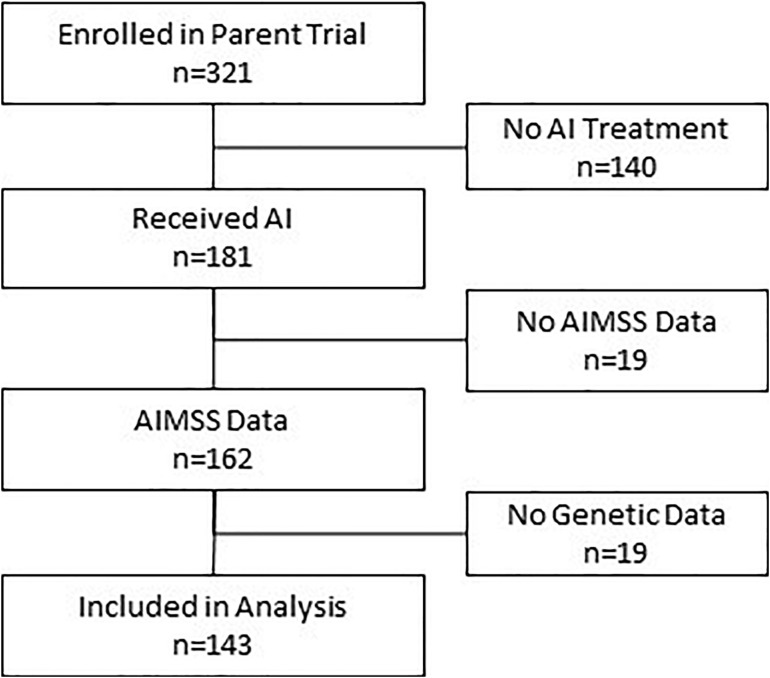
Consort diagram describing patient flow into study and analysis. Of the 321 patients enrolled in the observational clinical study, 143 had all required data and were included in this analysis.

The 143 AI-treated patients who completed the PRO measures assessing AIMSS at baseline and on-treatment at 3 and/or 6 months and with genetic information were included in this analysis (Figure 1). The patients were mostly white (85%) with a median age of 67 years (Table 1). Anastrozole (78%) and letrozole (20%) were used more frequently than exemestane (2%). AIMSS data from the PROMIS pain interference and physical function questionnaires and from the joint pain question on the FACT-ES were available for 99 and 90% of patients at 3 and 6 months, respectively. AIMSS were experienced by 31, 26, or 53% of patients when defined by the pain interference, physical function, or joint pain criteria, respectively. All genotypes were successfully determined in all patients, and all genetic data passed standard quality control including assessment of call rate. All variant distributions were in Hardy–Weinberg equilibrium (Hardy, 1908; Deng et al., 2001) in the entire genotyped cohort (*p* > 0.05) except *VDR* rs11568820, which was within expected proportions in the self-reported White cohort (*p* = 0.61) indicating the presence of population admixture (Early Breast Cancer Trialists’ Collaborative Group, 2015). The number of variant allele carriers is reported in Table 2.

**TABLE 1:** Clinical information of subjects included in the analysis.

Characteristic		*n* = 143
Race	White	122 (85%)
	Black	15 (11%)
	Other/Unknown	6 (4%)
Age	Years	67.0 (47.0–86.0)
Body mass index	kg/m^2^	27.8 (19.1–45.3)
Aromatase inhibitor	Anastrozole	112 (78%)
	Letrozole	28 (20%)
	Exemestane	3 (2%)
Prior chemotherapy	Taxane chemotherapy	38 (27%)
	Non-taxane or no chemotherapy	105 (73%)
On-treatment PRO questionnaires Completed	3 and 6 months	127 (89%)
	Only 3 months	14 (10%)
	Only 6 months	2 (1%)
Pain Interference	≥4 point increase*	45 (31%)
Physical Function	≥4 point decrease*	37 (26%)
Joint pain	≥1 point increase**	74 (52%)

*^∗^PROMIS T-score change from baseline to 3 and/or 6 months. ^∗∗^FACT-ES score change from baseline to 3 and/or 6 months for item, “I have pain in my joints.” Numbers presented as n (%) or median (range).*

**TABLE 2:** Unadjusted genetic associations for each variant with AIMSS endpoints.

				Pain interference^*a*^			Physical function^*b*^			Joint pain^*c*^		
SNP	Gene	Risk effect^*c*^	Carriers^*d*^	OR	95% CI	*p*-value^*e*^	OR	95% CI	*p*-value^*e*^	OR	95% CI	*p*-value^*e*^
rs11849538^*f*^	*TCL1A*	Higher	30	1.29	0.55, 3.07	0.56	1.29	0.52, 3.17	0.59	0.73	0.30, 1.75	0.48
rs2073618	*OPG*	Higher	102	1.36	0.60, 3.10	0.46	0.94	0.41, 2.16	0.88	**3.33**	**1.48, 7.49**	**0.0036**
rs7984870	*RANKL*	Higher	91	0.99	0.46, 2.10	0.98	0.98	0.44, 2.18	0.97	0.67	0.32, 1.42	0.30
rs11568820	*VDR*	Higher	64	0.94	0.46, 1.95	0.88	0.85	0.39, 1.82	0.67	0.51	0.25, 1.05	0.068
rs2234693	*ESR1*	Higher	106	1.46	0.63, 3.39	0.38	1.07	0.46, 2.51	0.87	0.97	0.44, 2.15	0.94
rs9322336	*ESR1*	Higher	37	1.04	0.46, 2.36	0.92	1.04	0.44, 2.44	0.93	1.45	0.64, 3.26	0.38
rs9340799	*ESR1*	Lower	89	1.10	0.52, 2.31	0.81	1.14	0.52, 2.50	0.74	1.08	0.52, 2.22	0.84
rs10046	*CYP19A1*	Lower	99	0.67	0.31, 1.46	0.32	0.92	0.41, 2.07	0.83	1.19	0.55, 2.57	0.65
rs934635	*CYP19A1*	Higher	38	1.32	0.58, 2.99	0.50	1.59	0.69, 3.68	0.28	0.96	0.43, 2.15	0.93
rs16964189	*CYP19A1*	Lower	61	1.67	0.80, 3.47	0.17	1.09	0.51, 2.36	0.82	0.96	0.47, 1.97	0.91
rs4646536	*CYP27B1*	Higher	75	0.89	0.43, 1.83	0.74	0.91	0.43, 1.95	0.81	1.44	0.71, 2.93	0.31
rs6163	*CYP17A1*	Lower	94	1.49	0.68, 3.24	0.32	0.74	0.34, 1.63	0.46	1.05	0.51, 2.19	0.89
rs7176005	NA/Chr15	Lower	40	1.03	0.46, 2.30	0.94	1.34	0.59, 3.07	0.48	0.74	0.34, 1.64	0.46

*OR: Odds ratio, 95% CI: 95% Confidence interval. Bold denotes *p* < 0.05 in unadjusted analysis. ^*a*^ ≥4-point increase PROMIS T-score from baseline to 3 and/or 6 months. ^*b*^ ≥4-point decrease in PROMIS T-score from baseline to 3 and/or 6 months. ^*c*^ ≥1-point increase on FACT-ES item, “I have pain in my joints” from baseline to 3 and/or 6 months. ^*c*^Prespecified expected direction of effect on AIMSS risk in patients carrying the variant allele compared to the homozygous wild-type. ^*d*^Number of individuals who were heterozygous or homozygous carriers of the variant allele. ^*e*^*p*-values are unadjusted for multiple comparisons. ^*f*^Prespecified primary hypothesis was that carriers of *TCL1A* rs11849538 had greater risk of AIMSS, as defined by the pain interference endpoint.*

### Genetic Associations With AIMSS

**FIGURE 2:**
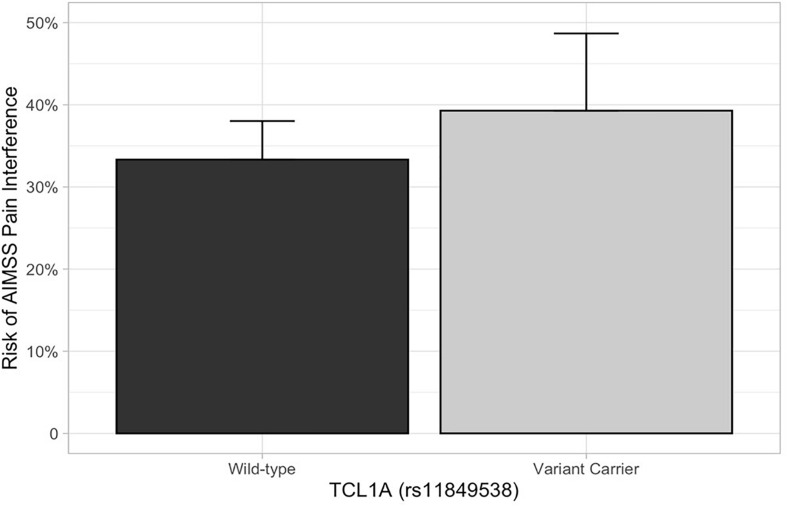
AIMSS risk stratified by *TCL1A* rs11849538. The proportion of patients who experienced AIMSS (*Y*-axis), as defined by a ≥ 4-point increase in PROMIS T-score from baseline to 3 or 6 months, is indicated by the box (standard error indicated by the vertical line). In the primary analysis, there was no increased AIMSS risk, as defined by an increase in the pain interference score from baseline to 3 or 6 months by at least four points, in patients carrying *TCL1A* rs11849538 (*n* = 30, unadjusted odds ratio = 1.29, 95% confidence interval: 0.55–3.07, *p* = 0.56).

**FIGURE 3:**
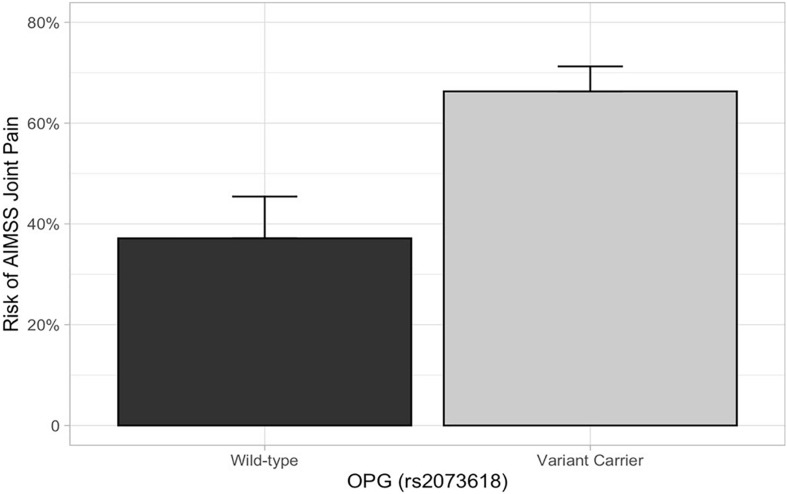
AIMSS risk stratified by *OPG* rs2073618. The proportion of patients who experienced AIMSS (*Y*-axis), as defined by a ≥ 1 point increase in FACT-ES from baseline to 3 or 6 months, is indicated by the box (standard error indicated by the vertical line). In the secondary analysis, patients carrying *OPG* rs2073618 (*n* = 102) had a greater risk of AIMSS, defined by the increase in joint pain measured by the FACT-ES (unadjusted OR = 3.33, 95% CI: 1.48–7.49, *p* = 0.004).

In the primary analysis, patients carrying *TCL1A* rs11849538 did not have increased risk of AIMSS, as defined by the primary endpoint of the increased pain interference T-score from baseline to 3 or 6 months by at least four points (unadjusted odds ratio (OR) = 1.29, 95% confidence interval (95% CI): 0.55–3.07, *p* = 0.56, Table 2 and Figure 2). None of the 12 secondary genetic predictors were associated with AIMSS as defined by the increase in the pain interference T-score (all *p* > 0.05). In the secondary analyses of the increase in joint pain as measured by the single question on the FACT-ES questionnaire, only *OPG* rs2073618 was associated with AIMSS in the expected direction of increasing risk (unadjusted OR = 3.33, 95% CI: 1.48–7.49, *p* = 0.004, Figure 3). This association persisted after adjustment for relevant covariates (adjusted OR = 3.98, 95% CI: 1.61–9.84, *p* = 0.003, Table 3), none of which had significant univariate associations with AIMSS in this relatively small cohort (all *p* > 0.05). This association would also be significant if a Bonferroni correction were applied to the secondary analyses (0.05/12 = 0.0042). In *post hoc* analyses, the effect of *OPG* rs2073618 could also be explained by an additive genetic model [OR = 2.06 (1.22–3.50), *p* = 0.007]. None of the other secondary analyses had significant univariate associations in the expected direction (Table 2).

**TABLE 3:** Multivariable association of *OPG* rs2073618 with worsening of joint pain measured on FACT-ES.

Variable	OR	95% CI	*p*-value
*OPG* rs2073618 (dominant model)	3.98	1.61, 9.84	0.003
Age	0.97	0.91, 1.02	0.21
White Race (vs. other)	0.70	0.21, 2.27	0.55
BMI	1.04	0.97, 1.13	0.25
Taxane Chemotherapy (vs. non-taxane or no chemotherapy)	0.94	0.39, 2.30	0.90
Anastrozole (vs. other AI)	0.54	0.22, 1.36	0.19

*BMI, Body mass index; AI, aromatase inhibitor; OR, odds ratio; 95% CI, 95% confidence interval.*

## Discussion

AI-induced musculoskeletal symptoms are one of the most common adverse effects of adjuvant AI treatment and are a primary reason for early discontinuation of adjuvant endocrine therapy, a treatment that reduces risks of recurrence and improves survival (Baum et al., 2003; Felson and Cummings, 2005; Coates et al., 2007; Partridge et al., 2008; Mao et al., 2009; Hershman et al., 2011; Murphy et al., 2012; Hadji et al., 2014; Moscetti et al., 2015; Olufade et al., 2015; Basch et al., 2017; Beckwée et al., 2017; Gupta et al., 2020). The objective of this study was to replicate associations with AIMSS risk in an independent patient cohort for SNPs that have been previously associated with AIMSS. Although we could not successfully replicate an increased AIMSS risk for carriers of *TCL1A* rs11849538, we demonstrated that patients who carried *OPG* rs2073618 had increased AIMSS risk.

Our finding that patients who carried the *OPG* rs2073618 variant G allele have increased AIMSS risk is consistent with several prior retrospective pharmacogenetic analyses. The rs2073618 variant is a C > G substitution with a minor allele frequency close to 0.5 in Caucasians, making it possible that researchers could inadvertently swap the wild-type and variant alleles depending on which strand was genotyped (the allele frequencies for this variant and all other variants in this analysis can be viewed in dbSNP^1^). Therefore, the resulting amino acid change for this missense variant (Asp3Lysine or N3K) can be used to definitively refer to the patient’s phenotype. In our study, we genotyped the *OPG* forward strand where a wild-type C allele results in AAC (asparagine) and a variant G allele results in AAG (lysine). Wang et al. initially hypothesized that SNPs in OPG, which encodes osteoprotegerin, could be associated with risk of AIMSS. Using a large cohort (*n* = 420) of AI-treated patients, these investigators found that patients who carried the allele encoding lysine had lower OPG expression and higher AIMSS risk (Dempsey et al., 2018). They also found that carriers of this variant had greater levels of the bone turnover markers carboxy terminal telopeptide and procollagen type I N-terminal propeptide and greater reduction in bone mineral density at the lumbar spine. Wang et al. also summarized the prior evidence that this SNP is associated with many bone-related conditions, likely due to enhanced bone resorption. This association was then partially replicated in a smaller (*n* = 154) cohort of AI-treated patients, in which rs2073618 variant (lysine) carriers reported greater musculoskeletal symptoms and pain severity (dominant *p* = 0.046). Interestingly, in this previous study, the increased AIMSS risk was restricted to the heterozygous carriers (Mao et al., 2011; Wang et al., 2013). In our cohort, the risk of AIMSS was greater in patients who were heterozygous (40/62 = 65%) or homozygous (21/30 = 70%) lysine carriers compared with homozygous asparagine patients (13/35 = 37%), consistent with an additive or dominant genetic effect. However, not all studies have successfully replicated this association, including our prior replication attempt in the ELPh cohort where we were unable to detect an association of rs2073618, perhaps due to the use of a different AIMSS endpoint defined by a musculoskeletal symptom cluster (Lintermans et al., 2016). Our current analysis supports the initial discovery and replication attempts and further suggests that carriers of lysine at rs2073618 have increased AIMSS risk.

None of the other candidate SNPs that we selected for this replication study were associated with our AIMSS phenotypes. This includes *TCL1A* rs11849538, which was originally discovered in a genome-wide association study conducted in a nested case–control study of patients who experienced AIMSS while receiving anastrozole or letrozole on the MA.27 clinical trial (Liu et al., 2012). Previous attempts and this current study have not replicated this association in a cohort of patients treated with AIs (Park et al., 2011).

Our results indicate that *OPG* rs2073618 may be a risk factor for AIMSS. If successfully validated in additional breast cancer cohorts, this genetic variant could potentially be used to identify patients at risk for musculoskeletal toxicity during AI therapy for personalized treatment, symptom monitoring, and symptom management that could lead to improve breast cancer outcomes. The approach to personalizing treatment based on the presence of this variant will require further understanding of the risk status in variant carriers. If this variant only increases AIMSS risk from a specific AI, it could provide the rationale for choosing that AI over the others. Alternatively, if carriers of this variant have higher AIMSS risk regardless of which AI they receive, the presence of this variant may justify enhanced side-effect monitoring (Early Breast Cancer Trialists’ Collaborative Group Davies et al., 2011) and symptom management or consideration of alternative treatment options such as tamoxifen (Sheppard et al., 2020). Finally, rs2073618 is very common in Caucasians and Asians, with minor allele frequencies of ∼45 and ∼25% in these groups, respectively, which will be much more efficient for preemptive genetic testing compared to other low-frequency variants that require screening many patients to identify the small number of patients at risk. The potential clinical benefit of preemptive testing to guide personalized treatment selection would likely need to be demonstrated in prospective clinical trials prior to clinical translation.

This pharmacogenetic study was conducted in a prospectively accrued real-world clinic-based cohort of patients from whom treatment-related AIMSS data were collected via validated PRO outcome measures prior to and at predefined time points during treatment. This analysis used clinically relevant definitions of AIMSS, and all analyses were conducted with a predefined expected direction of effect. While these analytical decisions improved the likelihood that our finding is valid, the lack of statistical correction for the 38 unique secondary analyses conducted increases the chances that this was a false-positive replication and therefore additional validation studies are needed. Our study had several additional limitations that need to be considered. This cohort was relatively small for a retrospective pharmacogenetic analysis, which precludes further analyses to determine whether the effect is exclusive to a single AI or shared among two or all three of the agents. Although there is evidence that AIMSS is more common in African Americans (67) our cohort was insufficiently large or diverse (85% Caucasian) to conduct analyses within individual racial subcohorts. Additionally, this study was likely underpowered to detect associations for less common variants or those with smaller effect sizes, and we were not able to explore haplotypes for genes with multiple variants including *CYP19A1* and *ESR1*. Moreover, the PRO outcome measures used in this study were not specific to AIMSS. The PROMIS pain interference measure addresses pain of any type, and multiple factors, such as other medications or physical injuries, could contribute to pain interference or physical function limitations. Although we did not exclude patients taking other medications with musculoskeletal side effects or patients with preexisting musculoskeletal conditions from participation, the fact that we compared PRO data from 3 and/or 6 months to baseline makes it likely that that AIMSS we detected using the PRO measures are attributable to AI treatment.

In conclusion, in our secondary analyses of a prospectively accrued cohort of HR + patients with early-stage breast cancer treated with an adjuvant AI, patients carrying lysine at *OPG* rs2073618 had greater risk of treatment-related AIMSS. Further retrospective pharmacogenetic analyses are needed to validate this clinical association, and preclinical functional studies are needed to further validate the mechanisms underlying this association. Upon validation, prospective genotyped-guided treatment trials are necessary to demonstrate that preemptive genotyping of rs2073618 can improve clinical outcomes in patients with HR + early-stage breast cancer receiving adjuvant endocrine treatment by guiding enhanced symptom monitoring and symptom management interventions with the goal of improving treatment adherence and persistence.

## Data Availability Statement

The raw data supporting the conclusions of this article will be made available by the authors, without undue reservation.

## Ethics Statement

The studies involving human participants were reviewed and approved by the Johns Hopkins IRB. The patients/participants provided their written informed consent to participate in this study.

## Author Contributions

DH, KS, JR, and VS contributed to conception and design of the study. YZ and KK conducted the data analysis. CG and AP conducted the genetic analysis. JL, AB, and VS enrolled participants. DH wrote the original draft of the manuscript, which was subsequently reviewed and edited by all co-authors. All authors contributed to the article and approved the submitted version.

## Conflict of Interest

KS research funding from Pfizer. Spouse with stock in Abbvie and ABT Labs. NH received research grants to institution from Pfizer. VS received research grants to institution from Abbvie, Biocept, Pfizer, Novartis, and Puma Biotechnology. Member, Data Safety Monitoring Board, Immunomedics, Inc. The remaining authors declare that the research was conducted in the absence of any commercial or financial relationships that could be construed as a potential conflict of interest.

## Footnotes

1 https://www.ncbi.nlm.nih.gov/snp/rs2073618

## References

[B1] Basch E. Deal A. M. Dueck A. C. Scher H. I. Kris M. G. Hudis C. (2017). Overall survival results of a trial assessing patient-reported outcomes for symptom monitoring during routine cancer treatment. *JAMA* 318 197–198. 10.1001/jama.2017.7156 28586821 PMC5817466

[B2] Baum M. Buzdar A. Cuzick J. Forbes J. Houghton J. Howell A. (2003). Anastrozole alone or in combination with tamoxifen versus tamoxifen alone for adjuvant treatment of postmenopausal women with early-stage breast cancer: results of the ATAC (Arimidex, Tamoxifen Alone or in Combination) trial efficacy and safety update analyses. *Cancer* 98 1802–1810. 10.002/cncr.11745 14584060

[B3] Beckwée D. Leysen L. Meuwis K. Adriaenssens N. (2017). Prevalence of aromatase inhibitor-induced arthralgia in breast cancer: a systematic review and meta-analysis. *Support. Care Cancer* 25 1673–1686. 10.007/s00520-017-3613-z 28204994

[B4] Bender C. M. Gentry A. L. Brufsky A. M. Casillo F. E. Cohen S. M. Dailey M. M. (2014). Influence of patient and treatment factors on adherence to adjuvant endocrine therapy in breast cancer. *Oncol. Nurs. Forum* 41 274–285. 10.1188/14.ONF.274-285 24769592 PMC4090095

[B5] Chim K. Xie S. X. Stricker C. T. Li Q. S. Gross R. Farrar J. T. (2013). Joint pain severity predicts premature discontinuation of aromatase inhibitors in breast cancer survivors. *BMC Cancer* 13:401. 10.1186/471-2407-13-401 PMC3847371 24004677

[B6] Coates A. S. Keshaviah A. Thürlimann B. Mouridsen H. Mauriac L. Forbes J. F. (2007). Five years of letrozole compared with tamoxifen as initial adjuvant therapy for postmenopausal women with endocrine-responsive early breast cancer: update of study BIG 1-98. *J. Clin. Oncol.* 25 486–492. 10.1200/JCO.2006.08.8617 17200148

[B7] Crew K. D. Greenlee H. Capodice J. Raptis G. Brafman L. Fuentes D. (2007). Prevalence of joint symptoms in postmenopausal women taking aromatase inhibitors for early-stage breast cancer. *J. Clin. Oncol.* 25 3877–3883. 10.1200/JCO.2007.10.7573 17761973

[B8] Dempsey J. M. Xi J. Henry N. L. Rae J. M. Hertz D. L. (2018). Attempted replication of SNPs in RANKL and OPG with musculoskeletal adverse events during aromatase inhibitor treatment for breast cancer. *Physiol. Genomics* 50 98–99. 10.1152/physiolgenomics.00085.2017 29212847 PMC5867615

[B9] Deng H. W. Chen W. M. Recker R. R. (2001). Population admixture: detection by Hardy-Weinberg test and its quantitative effects on linkage-disequilibrium methods for localizing genes underlying complex traits. *Genetics* 157 885–897. 10.1093/genetics/157.2.885 11157005 PMC1461540

[B10] Early Breast Cancer Trialists’ Collaborative Group. (2015). Aromatase inhibitors versus tamoxifen in early breast cancer: patient-level meta-analysis of the randomised trials. *Lancet* 386 1341–1352. 10.1016/S0140-6736(15)61074-1 26211827

[B11] Early Breast Cancer Trialists’ Collaborative Group Davies C. Godwin J. Gray R. Clarke M. Cutter D. (2011). Relevance of breast cancer hormone receptors and other factors to the efficacy of adjuvant tamoxifen: patient-level meta-analysis of randomised trials. *Lancet* 378 771–784. 10.1016/S0140-6736(11)60993-8 21802721 PMC3163848

[B12] Fallowfield L. J. Leaity S. K. Howell A. Benson S. Cella D. (1999). Assessment of quality of life in women undergoing hormonal therapy for breast cancer: validation of an endocrine symptom subscale for the FACT-B. *Breast Cancer Res. Treat.* 55 189–199. 10481946 10.1023/a:1006263818115

[B13] Felson D. T. Cummings S. R. (2005). Aromatase inhibitors and the syndrome of arthralgias with estrogen deprivation. *Arthritis Rheumatism* 52 2594–2598. 10.1002/art.21364 16142740

[B14] Fontein D. B. Houtsma D. Nortier J. W. Baak-Pablo R. F. Kranenbarg E. M. van der Straaten T. R. (2014). Germline variants in the CYP19A1 gene are related to specific adverse events in aromatase inhibitor users: a substudy of Dutch patients in the TEAM trial. *Breast Cancer Res. Treat.* 144 599–606. 10.1007/s10549-014-2873-2 24590773

[B15] Francis P. A. Pagani O. Fleming G. F. Walley B. A. Colleoni M. Láng I. (2018). Tailoring adjuvant endocrine therapy for premenopausal breast cancer. *N. Engl. J. Med.* 379 122–137. 10.1056/NEJMoa1803164 29863451 PMC6193457

[B16] Garcia S. F. Cella D. Clauser S. B. Flynn K. E. Lad T. Lai J. S. (2007). Standardizing patient-reported outcomes assessment in cancer clinical trials: a patient-reported outcomes measurement information system initiative. *J. Clin. Oncol.* 25 5106–5112. 10.1200/jco.2007.12.2341 17991929

[B17] Garcia-Giralt N. Rodriguez-Sanz M. Prieto-Alhambra D. Servitja S. Torres-Del Pliego E. Balcells S. (2013). Genetic determinants of aromatase inhibitor-related arthralgia: the B-ABLE cohort study. *Breast Cancer Res. Treat.* 140 385–395. 10.1007/s10549-013-2638-3 23868189

[B18] Gupta A. Henry N. L. Loprinzi C. L. (2020). Management of aromatase inhibitor-induced musculoskeletal symptoms. *Oncol. Pract.* 16 733–739. 10.1200/OP.20.00113 32780640

[B19] Hadji P. Jackisch C. Bolten W. Blettner M. Hindenburg H. J. Klein P. (2014). COMPliance and arthralgia in clinical therapy: the COMPACT trial, assessing the incidence of arthralgia, and compliance within the first year of adjuvant anastrozole therapy. *Ann. Oncol.* 25 372–377. 10.1093/annonc/mdt513 24355487

[B20] Hadji P. Ziller V. Kyvernitakis J. Bauer M. Haas G. Schmidt N. (2013). Persistence in patients with breast cancer treated with tamoxifen or aromatase inhibitors: a retrospective database analysis. *Breast Cancer Res. Treat.* 138 185–191. 10.1007/s10549-013-2417-1 23334803

[B21] Hardy G. H. (1908). Mendelian proportions in a mixed population. *Science* 28 49–50. 10.1126/science.28.706.49 17779291

[B22] Henry N. L. Azzouz F. Desta Z. Li L. Nguyen A. T. Lemler S. (2012). Predictors of aromatase inhibitor discontinuation as a result of treatment-emergent symptoms in early-stage breast cancer. *J. Clin. Oncol.* 30 936–942. 10.1200/JCO.2011.38.0261 22331951 PMC3341106

[B23] Henry N. L. Skaar T. C. Dantzer J. Li L. Kidwell K. Gersch C. (2013). Genetic associations with toxicity-related discontinuation of aromatase inhibitor therapy for breast cancer. *Breast Cancer Res. Treat.* 138 807–816. 10.1007/s10549-013-2504-3 23546553 PMC3646626

[B24] Henry N. L. Speth K. Lintermans A. Kidwell K. M. Carlson R. Hayes D. F. (2017). Associations between patient and anthropometric characteristics and aromatase inhibitor discontinuation. *Clin. Breast Cancer* 17 350–355. 10.1016/j.clbc.2017.03.002 28365336 PMC5537010

[B25] Hershman D. L. Kushi L. H. Hillyer G. C. Coromilas E. Buono D. Lamerato L. (2016). Psychosocial factors related to non-persistence with adjuvant endocrine therapy among women with breast cancer: the breast cancer quality of care study (BQUAL). *Breast Cancer Res. Treat.* 157 133–143. 10.1007/s10549-016-3788-x 27086286 PMC4867255

[B26] Hershman D. L. Kushi L. H. Shao T. Buono D. Kershenbaum A. Tsai W.-Y. (2010). early discontinuation and nonadherence to adjuvant hormonal therapy in a cohort of 8,769 early-stage breast cancer patients. *J. Clin. Oncol.* 28 4120–4128. 10.1200/JCO.2009.25.9655 20585090 PMC2953970

[B27] Hershman D. L. Shao T. Kushi L. H. Buono D. Tsai W. Y. Fehrenbacher L. (2011). Early discontinuation and non-adherence to adjuvant hormonal therapy are associated with increased mortality in women with breast cancer. *Breast Cancer Res. Treat.* 126 529–537. 10.1007/s10549-010-1132-4 20803066 PMC3462663

[B28] Hershman D. L. Unger J. M. Hillyer G. C. Moseley A. Arnold K. B. Dakhil S. R. (2020). Randomized trial of text messaging to reduce early discontinuation of adjuvant aromatase inhibitor therapy in women with early-stage breast cancer: SWOG S1105. *J. Clin. Oncol.* 38 2122–2129. 10.1200/JCO.19.02699 32369401 PMC7325363

[B29] Hertz D. L. Henry N. L. Rae J. M. (2017). Germline genetic predictors of aromatase inhibitor concentrations, estrogen suppression and drug efficacy and toxicity in breast cancer patients. *Pharmacogenomics* 18 481–499. 10.2217/pgs-2016-0205 28346074 PMC6219438

[B30] Ingle J. N. Schaid D. J. Goss P. E. Liu M. Mushiroda T. Chapman J. A. (2010). Genome-wide associations and functional genomic studies of musculoskeletal adverse events in women receiving aromatase inhibitors. *J. Clin. Oncol.* 28 4674–4682. 10.1200/JCO.2010.28.5064 20876420 PMC3020700

[B31] Jensen R. E. Moinpour C. M. Potosky A. L. Lobo T. Hahn E. A. Hays R. D. (2017a). Responsiveness of 8 patient-reported outcomes measurement information system (PROMIS) measures in a large, community-based cancer study cohort. *Cancer* 123 327–335. 10.1002/cncr.30354 27696377 PMC5222745

[B32] Jensen R. E. Potosky A. L. Moinpour C. M. Lobo T. Cella D. Hahn E. A. (2017b). United States population-based estimates of patient-reported outcomes measurement information system symptom and functional status reference values for individuals with cancer. *J. Clin. Oncol.* 35 1913–1920. 10.1200/jco.2016.71.4410 28426375 PMC5466008

[B33] Kadakia K. C. Snyder C. F. Kidwell K. M. Seewald N. J. Flockhart D. A. Skaar T. C. (2016). Patient-reported outcomes and early discontinuation in aromatase inhibitor-treated postmenopausal women with early stage breast cancer. *Oncologist* 21 539–546. 10.1634/theoncologist.2015-0349 27009936 PMC4861358

[B34] Kemp A. Preen D. B. Saunders C. Boyle F. Bulsara M. Malacova E. (2014). Early discontinuation of endocrine therapy for breast cancer: who is at risk in clinical practice? *Springerplus* 3:282. 10.1186/2193-1801-3-282 24936397 PMC4058005

[B35] Kidwell K. M. Harte S. E. Hayes D. F. Storniolo A. M. Carpenter J. Flockhart D. A. (2014). Patient-reported symptoms and discontinuation of adjuvant aromatase inhibitor therapy. *Cancer* 120 2403–2411. 10.1002/cncr.28756 24802413 PMC4126845

[B36] Kuba S. Ishida M. Nakamura Y. Taguchi K. Ohno S. (2016). Persistence and discontinuation of adjuvant endocrine therapy in women with breast cancer. *Breast Cancer* 23 128–133. 10.1007/s12282-014-0540-4 24934610

[B37] Leyland-Jones B. Gray K. P. Abramovitz M. Bouzyk M. Young B. Long B. (2015). CYP19A1 polymorphisms and clinical outcomes in postmenopausal women with hormone receptor-positive breast cancer in the BIG 1-98 trial. *Breast Cancer Res. Treat.* 151 373–384. 10.1007/s10549-015-3378-3 25935582 PMC4763278

[B38] Lintermans A. Van Asten K. Jongen L. Van Brussel T. Laenen A. Verhaeghe J. (2016). Genetic variant in the osteoprotegerin gene is associated with aromatase inhibitor-related musculoskeletal toxicity in breast cancer patients. *Eur. J. Cancer* 56 31–36. 10.1016/j.ejca.2015.12.013 26798969

[B39] Liu M. Wang L. Bongartz T. Hawse J. R. Markovic S. N. Schaid D. J. (2012). Aromatase inhibitors, estrogens and musculoskeletal pain: estrogen-dependent T-cell leukemia 1A (TCL1A) gene-mediated regulation of cytokine expression. *Breast Cancer Res.* 14:R41. 10.1186/bcr3137 22405131 PMC3446375

[B40] Mao J. J. Stricker C. Bruner D. Xie S. Bowman M. A. Farrar J. T. (2009). Patterns and risk factors associated with aromatase inhibitor-related arthralgia among breast cancer survivors. *Cancer* 115 3631–3639. 10.1002/cncr.24419 19517460 PMC3569524

[B41] Mao J. J. Su H. I. Feng R. Donelson M. L. Aplenc R. Rebbeck T. R. (2011). Association of functional polymorphisms in CYP19A1 with aromatase inhibitor associated arthralgia in breast cancer survivors. *Breast Cancer Res.* 13:R8. 10.1186/bcr2813 21251330 PMC3109575

[B42] Moscetti L. Agnese Fabbri M. Sperduti I. Fabrizio N. Frittelli P. Massari A. (2015). Adjuvant aromatase inhibitor therapy in early breast cancer: what factors lead patients to discontinue treatment? *Tumori* 101 469–473. 10.5301/tj.5000376 26108239

[B43] Murphy C. C. Bartholomew L. K. Carpentier M. Y. Bluethmann S. M. Vernon S. W. (2012). Adherence to adjuvant hormonal therapy among breast cancer survivors in clinical practice: a systematic review. *Breast Cancer Res. Treat.* 134 459–478. 10.1007/s10549-012-2114-5 22689091 PMC3607286

[B44] Nabieva N. Fehm T. Häberle L. de Waal J. Rezai M. Baier B. (2018a). Influence of side-effects on early therapy persistence with letrozole in post-menopausal patients with early breast cancer: results of the prospective Evaluate-TM study. *Eur. J. Cancer* 96 82–90. 10.1016/j.ejca.2018.03.020 29679775

[B45] Nabieva N. Kellner S. Fehm T. Häberle L. de Waal J. Rezai M. (2018b). Influence of patient and tumor characteristics on early therapy persistence with letrozole in postmenopausal women with early breast cancer: results of the prospective Evaluate-TM study with 3941 patients. *Ann. Oncol.* 29 186–192. 10.1093/annonc/mdx630 29045642

[B46] Neugut A. I. Zhong X. Wright J. D. Accordino M. Yang J. Hershman D. L. (2016). Nonadherence to medications for chronic conditions and nonadherence to adjuvant hormonal therapy in women with breast cancer. *JAMA Oncol.* 2 1326–1332. 10.001/jamaoncol.2016.1291 27281650

[B47] Olufade T. Gallicchio L. MacDonald R. Helzlsouer K. J. (2015). Musculoskeletal pain and health-related quality of life among breast cancer patients treated with aromatase inhibitors. *Support. Care Cancer* 23 447–455. 10.1007/s00520-014-2364-3 25128067

[B48] Park I. H. Lee Y. S. Lee K. S. Kim S. Y. Hong S. H. Jeong J. (2011). Single nucleotide polymorphisms of CYP19A1 predict clinical outcomes and adverse events associated with letrozole in patients with metastatic breast cancer. *Cancer Chemother. Pharmacol.* 68 1263–1271. 10.1007/s00280-011-1615-y 21442439

[B49] Partridge A. H. LaFountain A. Mayer E. Taylor B. S. Winer E. Asnis-Alibozek A. (2008). Adherence to initial adjuvant anastrozole therapy among women with early-stage breast cancer. *J. Clin. Oncol.* 26 556–562. 10.1200/JCO.2007.11.5451 18180462

[B50] Partridge A. H. Wang P. S. Winer E. P. Avorn J. (2003). Nonadherence to adjuvant tamoxifen therapy in women with primary breast cancer. *J. Clin. Oncol.* 21 602–606. 10.1200/JCO.2003.07.071 12586795

[B51] Rohlfs R. V. Weir B. S. (2008). Distributions of hardy-weinberg equilibrium test statistics. *Genetics* 180 1609–1616. 10.1534/genetics.108.088005 18791257 PMC2581961

[B52] Schalet B. D. Pilkonis P. A. Yu L. Dodds N. Johnston K. L. Yount S. (2016). Clinical validity of PROMIS depression, anxiety, and anger across diverse clinical samples. *J. Clin. Epidemiol.* 73 119–127. 10.1016/j.jclinepi.2015.08.036 26931289 PMC4928679

[B53] Sestak I. Cuzick J. Sapunar F. Eastell R. Forbes J. F. Bianco A. R. (2008). Risk factors for joint symptoms in patients enrolled in the ATAC trial: a retrospective, exploratory analysis. *Lancet Oncol.* 9 866–872. 10.1016/S470-2045(08)70182-7 18703382

[B54] Sheppard V. B. Dash C. Nomura S. Sutton A. L. Franco R. L. Lucas A. (2020). Physical activity, health-related quality of life, and adjuvant endocrine therapy-related symptoms in women with hormone receptor-positive breast cancer. *Cancer* 126 4059–4066. 10.1002/cncr.33054 32614992 PMC8018708

[B55] Shinn E. H. Broderick G. Fellman B. Johnson A. Wieland E. Moulder S. (2019). Simulating time-dependent patterns of nonadherence by patients with breast cancer to adjuvant oral endocrine therapy. *Clin. Cancer Inform.* 3 1–9. 10.1200/CCI.18.00091 31002563 PMC6873985

[B56] Sitlinger A. Shelby R. A. Van Denburg A. N. White H. Edmond S. N. Marcom P. K. (2019). Higher symptom burden is associated with lower function in women taking adjuvant endocrine therapy for breast cancer. *J. Geriatr. Oncol.* 10 317–321. 10.1016/j.jgo.2018.11.008 30553719 PMC6409104

[B57] Snyder C. F. Blackford A. L. Wolff A. C. Carducci M. A. Herman J. M. Wu A. W. (2013). Feasibility and value of patientviewpoint: a web system for patient-reported outcomes assessment in clinical practice. *Psychooncology* 22 895–901. 10.1002/pon.3087 22544513 PMC3415606

[B58] Snyder C. F. Jensen R. Courtin S. O. Wu A. W. (2009). PatientViewpoint: a website for patient-reported outcomes assessment. *Qual. Life Res.* 18 793–800. 10.1007/s11136-009-9497-8 19544089 PMC3073983

[B59] Teresi J. A. Ocepek-Welikson K. Cook K. F. Kleinman M. Ramirez M. Reid M. C. (2016). Measurement equivalence of the patient reported outcomes measurement information system(^®^) (PROMIS(^®^)) pain interference short form items: application to ethnically diverse cancer and palliative care populations. *Psychol. Test Assess. Model.* 58 309–352. 28983449 PMC5625836

[B60] Wagner L. I. Zhao F. Goss P. E. Chapman J. W. Shepherd L. E. Whelan T. J. (2018). Patient-reported predictors of early treatment discontinuation: treatment-related symptoms and health-related quality of life among postmenopausal women with primary breast cancer randomized to anastrozole or exemestane on NCIC Clinical Trials Group (CCTG) MA.27 (E1Z03). *Breast Cancer Res. Treat.* 169 537–548. 10.1007/s10549-018-4713-2 29455298 PMC6092930

[B61] Wang J. Lu K. Song Y. Xie L. Zhao S. Wang Y. (2013). Indications of clinical and genetic predictors for aromatase inhibitors related musculoskeletal adverse events in Chinese Han women with breast cancer. *PloS One* 8:e68798. 10.1371/journal.pone.0068798 23894347 PMC3716812

[B62] Wang J. Lu K. Song Y. Zhao S. Ma W. Xuan Q. (2015). RANKL and OPG polymorphisms are associated with aromatase inhibitor-related musculoskeletal adverse events in Chinese Han breast cancer patients. *PLoS One* 10:e0133964. 10.1371/journal.pone.0133964 26218592 PMC4547828

[B63] Wheeler S. B. Spencer J. Pinheiro L. C. Murphy C. C. Earp J. A. Carey L. (2019). Endocrine therapy nonadherence and discontinuation in black and white women. *J. Natl. Cancer Inst.* 111 498–508. 10.1093/jnci/djy136 30239824 PMC6510227

[B64] Wu A. W. White S. M. Blackford A. L. Wolff A. C. Carducci M. A. Herman J. M. (2016). Improving an electronic system for measuring PROs in routine oncology practice. *J. Cancer Surviv.* 10 573–582. 10.1007/s11764-015-0503-6 26644190 PMC4864116

[B65] Wulaningsih W. Garmo H. Ahlgren J. Holmberg L. Folkvaljon Y. Wigertz A. (2018). Determinants of non-adherence to adjuvant endocrine treatment in women with breast cancer: the role of comorbidity. *Breast Cancer Res. Treat.* 172 167–177. 10.1007/s10549-018-4890-z 30030708 PMC6208918

[B66] Yost K. J. Eton D. T. Garcia S. F. Cella D. (2011). Minimally important differences were estimated for six Patient-reported outcomes measurement information system-cancer scales in advanced-stage cancer patients. *J. Clin. Epidemiol.* 64 507–516. 10.1016/j.jclinepi.2010.11.018 21447427 PMC3076200

